# Supplementary material for: The divergent mitotic kinesin MKLP2 exhibits atypical structure and mechanochemistry
Source: eLife. 2017 Aug 11;6:e27793. doi: 10.7554/eLife.27793 (PMC5602324; doi:10.7554/eLife.27793)
Supplement: Transparent reporting form [file elife-27793-transrepform.docx]

***eLife’s* transparent reporting form**

We encourage authors to provide detailed information *within their submission* to facilitate the interpretation and replication of experiments. If you have any questions, please contact us: [editorial@elifesciences.org](mailto:editorial@elifesciences.org).

**Sample-size estimation**

- You should state whether an appropriate sample size was computed when the study was being designed
- You should state the statistical method of sample size computation and any required assumptions
- If no explicit power analysis was used, you should describe how you decided what sample (replicate) size (number) to use

Please outline where this information can be found within the submission (e.g., page numbers or figure legends), or explain why this information doesn’t apply to your submission:

Statistical methods were not used to predetermine sample size. Sample size and replicate number for the biochemical (p18, p19), and structural (p20) experiments were determined by available material, and the statistical significance of the conclusions were subsequently calculated according to the available data.

**Replicates**

- You should report how often each experiment was performed
- You should include a definition of biological versus technical replication
- The data obtained should be provided and sufficient information should be provided to indicate the number of independent biological and/or technical replicates
- If you encountered any outliers, you should describe how these were handled
- Criteria for exclusion/inclusion of data should be clearly stated
- High-throughput sequence data should be uploaded before submission, with a private link for reviewers provided (these are available from both GEO and ArrayExpress)

Please outline where this information can be found within the submission (e.g., page numbers or figure legends), or explain why this information doesn’t apply to your submission:

ATPase measurements were performed in duplicate on the same day (technical replicate; p18). No outliers were observed. For kinetic experiments, data for rate constant versus [mant ATP] was collected in duplicate (technical replicate; p18). Corresponding data for rate constant versus [mant ADP] were collected once in the presence and absence of microtubules (MTs) on the same day (p18). The absence of any appreciable outliers supports the accuracy of the measurements.

For the MT cosedimentation assay, the MKLP2 protein construct 25-520 in ADP, NN, and ADP.AlFx nucleotide states was incubated with MT at 14 different concentrations (14 data points); and in AMPPNP state it was incubated with 7 data points. Two technical replicates for 7 data points of the NN- and ADP- states were performed to confirm the observed unusual affinity for the ADP state. Where technical replicates were performed, the average and SEM are plotted, otherwise individual data points are shown (Legend to Figure 1, p22). An obvious outlier data point in ADP.AlFx state (at MT concentration = 0.75 μM), which has a spiked %MKLP2 in pellet much higher than points before and after it and likely due to errors from gel loading and/or sample preparation, was removed from the curve.

For time-resolved anisotropy experiments, anisotropy decays were acquired from two independent preparations of protein (biological replicate; p19). For each biochemical condition assayed, a minimum of 5 independent samples were acquired. No outliers were observed.

Information on cryo-electron microscopy data sets can be found in the Materials and Methods section under ‘Cryo-EM data collection and data processing’ (p20). The ADPAlFx, ADP and NN datasets were collected from two separate sample preparations (technical replicates), while the AMPPNP dataset was collected from three separate sample preparations.

**Statistical reporting**

- Statistical analysis methods should be described and justified
- Raw data should be presented in figures whenever informative to do so (typically when N per group is less than 10)
- For each experiment, you should identify the statistical tests used, exact values of N, definitions of center, methods of multiple test correction, and dispersion and precision measures (e.g., mean, median, SD, SEM, confidence intervals; and, for the major substantive results, a measure of effect size (e.g., Pearson's r, Cohen's d)
- Report exact p-values wherever possible alongside the summary statistics and 95% confidence intervals. These should be reported for all key questions and not only when the p-value is less than 0.05.

Steady state ATPase, transient kinetic and MT cosedimentation data were subjected to linear least squares fitting (p17,18).

The reported resolutions of cryo-electron microscopy reconstructions were estimated from the FSCtrue value, calculated by the gold-standard noise-substitution test as described in the legend of Figure 2-figure supplement 1.

Time-resolved fluorescence anisotropy data were subjected to non-linear optimization to determine best fitting single exponential decay parameters. Data reported represent the mean of parameters from fits of replicate data +/- the standard error for the fit parameter (p19).

Please outline where this information can be found within the submission (e.g., page numbers or figure legends), or explain why this information doesn’t apply to your submission:

(For large datasets, or papers with a very large number of statistical tests, you may upload a single table file with tests, Ns, etc., with reference to page numbers in the manuscript.)

**Additional data files (“source data”)**

- We encourage you to upload relevant additional data files, such as numerical data that are represented as a graph in a figure, or as a summary table
- Where provided, these should be in the most useful format, and they can be uploaded as “Source data” files linked to a main figure or table
- Include model definition files including the full list of parameters used
- Include code used for data analysis (e.g., R, MatLab)
- Avoid stating that data files are “available upon request”

Please indicate the figures or tables for which source data files have been provided:

There are no such source data files associated with this manuscript.
